# The role of DNMT1/hsa-miR-124-3p/BCAT1 pathway in regulating growth and invasion of esophageal squamous cell carcinoma

**DOI:** 10.1186/s12885-019-5815-x

**Published:** 2019-06-21

**Authors:** Bo Zeng, Xin Zhang, Jingling Zhao, Zhewei Wei, Haoshuai Zhu, Minyi Fu, Dawei Zou, Yanfen Feng, Honghe Luo, Yiyan Lei

**Affiliations:** 1 Department of Thoracic Surgery, the First Affiliated Hospital, Sun Yat-sen University, Guangzhou, China; 2 Department of Burns, the First Affiliated Hospital, Sun Yat-sen University, Guangzhou, China; 3 Department of Gastrointestinal Surgery, the First Affiliated Hospital, Sun Yat-sen University, Guangzhou, China; 4 Surgical and anesthesia center, the First Affiliated Hospital, Sun Yat-sen University, Guangzhou, China; 5 State Key Laboratory of Oncology in South China, Collaborative Innovation Center for Cancer Medicine, Sun Yat-sen University Cancer Center, Guangzhou, China; 6 Department of Pathology, Sun Yat-sen University Cancer Center, Guangzhou, China; 7 Guangzhou, China

**Keywords:** Esophageal squamous cell carcinoma, Hsa-miR-124-3p, Branched chain amino acid transaminase 1, DNA methyltransferase 1

## Abstract

**Background:**

Esophageal squamous cell carcinoma (ESCC) is the major subtype of esophageal cancer with high aggressiveness and poor prognosis. There is an urgent need for understanding the molecular mechanism underlying the development and progression of ESCC.

**Methods:**

ESCC tissues and corresponding non-neoplastic tissues were collected. The expression and function of miR-124-3p and BCAT1 in two cell lines KYSE-150 and Eca109 were determined.

**Results:**

We show downregulation of miR-124-3p expression in ESCC tissues, which is highly correlated with proliferation and migration of ESCC cell lines KYSE-150 and Eca109. miR-124-3p show high correlation with TNM stage and differentiation grade. Furthermore, miR-124-3p directly targets mRNA 3’UTR region of BCAT1, which results in upregulation of BCAT1 expression as observed in ESCC tissues and cell lines. Also, our data indicates that BCAT1 high expression is strongly linked to the disease-free survival, tumor size, pathologic stage, T classification and differentiation grade. On the other hand, we clarified the upstream mechanism regulating miR-124-3p expression in ESCC, which involves in the hypermethylation-silencing regulation mediated by DNA methyltransferase 1(DNMT1), which is of high expression in ESCC tissues and cell lines in the present study. In addition, DNMT1 knockdown or inhibition of DNMT1 function contributes to downregulation of miR-124-3p and BCAT1 expression.

**Conclusions:**

Our study thus clarifies a new mechanism that DNMT1/miR-124/BCAT1 axis regulates the development and progression of ESCC.

## Background

Esophageal cancer is one of the most common cancers with high aggressiveness and poor prognosis [1]. Esophageal squamous cell carcinoma (ESCC) is the major subtype of esophageal cancer and is highly prevalent in China [2]. Despite substantial improvements in clinical diagnosis and therapeutics over the past two decades, the 5-year survival rate in patients with ESCC is less than 25% [3]. There is an urgent need for understanding the molecular mechanism underlying the development and progression of ESCC.

In recent years, cancers have been widely regarded to be associated with epigenetic modifications that is defined as a stable change in gene expression without modifications in DNA sequence [4–6]. DNA methylation is one of the best known epigenetic mechanisms in cancer epigenetics. DNA methylation involves in the addition of a methyl (CH3) group to DNA with DNA methyltransferases (DNMTs), thereby often modifying gene function through regulation of gene expression [7]. DNA hypermethylation in the promotor region of tumor suppressor genes has been widely shown in various cancers [8, 9]. Similarly, a recent study on ESCC also showed aberrant DNA methylation in the different genes including HOXB2, NEFL, SLC15A3, OBSL1 and miR-124 [10]. However, it is lack of evidence that these genes are essentially associated with ESCC progression. Among of these genes, miR-124 caught our attention in view of the finding that large number of microRNAs(MiRNAs) have been shown important roles in the development of different cancers [11]. In the previous studies, miR-124 has been shown to be associated with several cancers including ESCC [10, 12]. For instance, miR-124 might have effects on decreasing risks of ESCC in subgroups of Chinese elderly persons [13].

A microRNA is a small non-coding RNA molecule, which functions in the post-transcriptional regulation of gene expression by partially complementing with the 3′-untranslated regions (3’UTR) of specific mRNAs [14]. According to the prediction of bioinformatics software of Miranda, we showed that 5′-UTR regions of hsa-miR-124-3p, a mature sequence of human miR-124 precursor, could bind to 3′-UTR region of branched chain amino acid transaminase 1(BCAT1) gene, the enzyme that catalyzes branched-chain alpha-keto acids to branched-chain L-amino acids essential for cell growth [15, 16]. BCAT1 overexpression has been shown to be associated with several cancers including glioblastoma [17], nasopharyngeal carcinoma [18], ovarian cancer [19] and hepatocellular carcinoma [20].

These findings prompted us to investigate the role of DNMT/miR-124/BCAT1 axis in regulating development and progression of ESCC. With the study of loss-of-function and gain-of-function in DNMT/miR-124/BCAT1, we showed that downregulation of hsa-miR-124-3p in ESCC tissues and cells contributed to the upregulation of BCAT1 expression, which enhanced the proliferation and invasion of ESCC. Furthermore, the data demonstrated that hsa-miR-124-3p downregulation was associated with hypermethylation in its promotor region, which resulted from increased expression of DNMT1. Our study thus clarifies a new mechanism that DNMT1/miR-124/BCAT1 axis regulates the development and progression of ESCC. These findings suggest that inhibitors against the activity of DNMT1 and/or BCAT1 might be a novel strategy to inhibit development and progression of ESCC.

## Methods

### Patients and tissue samples

The primary ESCC tissues and adjacent normal tissues were collected from patients who underwent surgery the First Affiliated Hospital, Sun Yat-sen University. All patients have not received any therapy before surgery. The specimens were collected immediately after removing the resected esophagus, and stored at − 80 °C. The study was obtained the informed consent from all the participating patients and the approval from the Ethics Committee of the First Affiliated Hospital, Sun Yat-sen University.

### Cell culture, cell transfection and drug treatment

Human normal esophageal cell line HEEC (Het-1A, ATCC® CRL-2692™) was obtained from the America Type Culture Collection in May 2017, and two human esophageal carcinoma cell lines, KYSE-150 (TCHu236) and Eca-109 (TCHu69), were obtained from Shanghai Institute of Biological Sciences, Chinese Academy of Sciences in May 2017. These cell lines have been authenticated by short-tandem repeat analyses. They are free of mycoplasma contamination.

The cells were maintained in RPMI-1640 medium (Invitrogen, Carlsbad, CA, USA) supplemented with 10% FBS (Gibco, Grand Island, NY, USA) and 1% Penicillin-Streptomycin (10,000 U/mL) (Invitrogen). For cell transfection, siRNAs including DNMT1 siRNA, BCAT1 siRNA, scrambled siRNA (NC siRNA), miR-124-3p mimics and its negative control (NC mimics) were supplied by Ribobio Company (Guangzhou, China) and pcDNA-BCAT1(OE-BCAT1) or pcDNA-negative (OE-NC) were constructed using pcDNA3.1(+) vector. KYSE-150 and Eca-109 cells grown in 6-well cell plates (1 × 10^6^) were transfected with siRNA or pcDNA3.1vector using Lipofectamine 2000 (Invitrogen) according to the manufacturer’s instruction. After 6 h, the transfection solution was replaced by complete medium.

For drug treatment, KYSE-150 or Eca109 cells were treated with 5 μM 5-Azacitidine (5-Aza, Sigma-Aldrich, Saint Louis, MO, USA), which is the inhibitor of DNMT1.

### Real-time PCR

Total RNA from tissue samples and cells were extracted with RNAprep pure tissue kit (Tiangen Biotech Co., LTD., Beijing, China) and Rneasy Mini Kit (Qiagen, Germantown, MD, USA) respectively. The mRNA of DNMT1, miR-124-3p and BCAT1 were amplified by quantitative real-time RT-PCR. It was carried out using SuperScriptIII Platinum SYBR Green One-Step qRT-PCR kit (Invitrogen) by an ABI 7500 Fast real-time PCR instrument (Applied Biosystems, Foster City, CA, USA). It was carried out with the following procedures: 50 °C for 3 min, 95 °C for 5 min, followed by 35 cycles of 95 °C for 15 s, 60 °C for 30 s. The specific primers used in quantitative real-time RT-PCR were displayed as follows: DNMT1: forward, 5′-CCTAGCCCCAGGATTACAAGG-3′; reverse, 5′-ACTCATCCGATTTGGCTCTTTC-3′; BCAT1: forward: 5′- GAGCCTGGAAAGGTGGAACTG-3′; reverse, 5′- GCTGACACCCATTATCTACTGCT-3′; GAPDH: forward, 5′-TGTTCGTCATGGGTGTGAAC-3′; reverse, 5′-ATGGCATGGACTGTGGTCAT-3′; hsa-miR-124-3p: forward, 5′-ACACTC CAGCTGGGTAAGGCACGCGGTGAA-3′; reverse, 5′- CTCAACTGGTGTCGTGGAGTCGGCAATTCAGTTGAGGGCATTCAC-3; U6: forward, 5′-CTCGCTTCGGCAGCACA-3′; reverse, 5′-AACGCTTCACGAATTTGCGT-3′. The data were analyzed according to the comparative Ct method.

### Bisulfite sanger sequencing

Genomic DNA from ESCC tissues or adjacent normal tissues was extracted using QIAamp DNA Mini Kit (Qiagen) and bisulfite converted using an EpiTect Bisulfite Kit (QIAGEN). The hsa-miR-124-3p was enriched by PCR with Taq DNA Polymerase (Invitrogen) and primer designed using Pyrosequencing Assay Design Software (Qiagen). The PCR products were cloned into pGEMT vector, then subjected to Sanger sequencing. Five single molecules were sequenced for each sample.

### Methylation specific PCR (MSP)

The bisulfite converted DNAs were used as the templates for the MSP assay, with miR-124-3p primers that distinguish the methylated (M) from unmethylated (U) DNA: M forward: 5′-GTATTTGGGGGTTTATTTTTTGTC-3′; M reverse: 5′- GAAACCGACTCGAACTTACGTA-3′; U forward: 5′-GTATTTGGGGGTTTATTTTTTGTT-3′; U reverse: 5′-ATCAAAACCAACTCAAACTTACATA-3′. PCR amplification was conducted at 95 °C for 3 min, followed by 30 cycles of 94 °C for 1 min, 56 °C for 1 min, 72 °C for 30 s, and the elongation at 72 °C for 5 min. A plasmid containing methylated miR-124-3p sequence and water without DNA template were used as positive control and negative control, respectively. Finally, MSP products were analyzed using agarose gel electrophoresis.

### Prediction of hsa-miR-124-3p targets

The target of hsa-miR-124-3p was predicted with the online software Miranda, which is an algorithm for determining potential target sites of microRNA in genomic sequences. Briefly, hsa-miR-124-3p sequences were first scanned in all genomic DNA/RNA sequences. Then, the potential target sites were identified with a dynamic programming local alignment and the thermodynamic stability estimation of RNA duplexes. The detected target with energies less than an energy threshold was identified as potential target sites.

### Luciferase assay

The human BCAT1 3′-UTR DNA sequence that was predicted to interact with miR-124-3p and the mutant BCAT1 3′-UTR DNA sequence were amplified and inserted into psi-CHECK2 renilla/firefly dual-luciferase expression vector (Promega, Mullion, WI, USA), with the following primers: BCAT1-WT-1: 5′-CCGCTCGAGTTTGAAGGTCCTCCAAGTCCGTGT-3′ (forward) and 5′-ATTTGCGGCCGCCTAATGCTGCCACTGAGCTGACAAG-3′ (reverse). BCAT1-MUT-1: 5′-AAGACTGAGGGTCTTCGCATACATAGATCTTTGTATC-3′ (forward) and 5′-GATACAAAGATCTATGTATGCGAAGACCCTCAGTCTT-3′ (reverse). BCAT1-WT-2: 5′-CCGCTCGAGGAGGGTGATGACATACATTTACTGG-3′ (forward) and 5′-ATTTGCGGCCGCAACTTTTCAGAATCCCTAGTGGC-3′ (reverse). BCAT1-MUT-2: 5′-ATTCCATATACATATCGCATACATATTGTGATAATTT-3′ (forward) and 5′-AAATTATCACAATATGTATGCGATATGTATATGGAAT-3′ (reverse). KYSE-150 or Eca-109 cells were co-transfected with the BCAT1-WT/BCAT1-MUT reporter constructs, and miR-124-3p mimics/NC mimics with Lipofectamine 2000 (Invitrogen). Luciferase activities were determined after 48 h using the Dual-Luciferase reporter assay system (Promega) on Enspire. Data are presented as the ratio of renilla luciferase activity to firefly luciferase.

### Immunohistochemistry staining

ESCC tissue or adjacent normal tissue samples were fixed in 10% neutral buffered formalin (NBF). Thereafter, the tissues were paraffin-processed and embedded. The deparaffinized tissue sections (4 mm thick) were stained with antibody against BCAT1 (Santa Cruz, Dallas, Texas, USA) for immunohistochemical analysis. Images of immunohistochemistry staining were photographed under a light microscope (Leica, Wetzlar, Germany).

### Western blot analysis

Total cell proteins were extracted using the mammalian protein extraction reagent including halt protease inhibitor cocktail (Thermo Fisher Scientific, Waltham, MA, USA). The protein concentrations were measured by BCA protein assay kit (Thermo Fisher Scientific) and equal amount of proteins were subjected to SDS-PAGE, then transferred to polyvinylidene difluoride membranes (Millipore, Billerica, MA, USA). After blocked with 5% nonfat milk, the membranes were incubated with primary antibodies against DNMT1, BCAT1, and GAPDH (Santa Cruz), respectively, and followed by incubation with the HRP-conjugated secondary antibodies. The signals were detected using an enhanced chemiluminescence kit (GE Healthcare Life Sciences, Pittsburgh, PA, USA).

### Immunofluorescence staining

KYSE-150 or Eca-109 cells grown on coverslips were treated with miR-124-3p mimics/NC mimics or BCAT1 siRNA/NC siRNA for 48 h. Then the cells were fixed with 4% paraformaldehyde and permeabilized with 0.5% Triton X-100 in PBS. After blocked with 3% bovine serum albumin, cells were incubated with primary antibodies against DNMT1, BCAT1 and DAPI. Subsequently, cells were stained with the Alexa Fluor 488-conjugated and FITC-conjugated secondary antibodies (BD Biosciences, San Diego, CA, USA). Finally, immunofluorescence images were visualized under LSM 510 Meta confocal microscope (Carl Zeiss GmbH, Oberkochen, Germany).

### CCK-8 assay

Cell viability was measured using Cell Counting Kit-8 (CCK-8) (Dojindo, Kumamoto, Japan). KYSE-150 or Eca-109 cells grown in 96-well plates were treated with miR-124-3p mimics/NC mimics or BCAT1 siRNA/NC siRNA for 24 h, 48 h or 72 h. After washing with PBS for three times. A 1:10 diluted CCK-8 solution was added and incubated at 37 °C for 2 h. The absorbance was determined at 450 nm on Enspire (PerkinElmer, Waltham, MA, USA).

### Colony formation assay

For colony formation assay, KYSE-150 or Eca-109 cells treated with miR-124-3p mimics/NC mimics or BCAT1 siRNA/NC siRNA were seeded in a 3 mm^2^ plate. After 2 weeks of incubation, plates were washed with PBS gently and stained with 0.5% crystal violet in 20% ethanol for 15 min at room temperature. Lastly, the cells were imaged after rinsed with PBS.

### Transwell assay

The invasion capability of cells was determined by the transwell assay. After transfection for 48 h, KYSE-150 or Eca-109 cells in serum-free media were transferred into the upper chamber of Transwell assay inserts (Millipore) and media supplemented 10% FBS was added to the bottom chamber. After incubation at 37 °C for 24 h, the invading cells through the membrane were stained with 0.5% crystal violet in 20% ethanol and photographed under the light microscope.

## Results

### Expression of hsa-miR-124-3p is down-regulated and its gene shows hypermethylation in ESCC tissues and cells

**Fig. 1:**
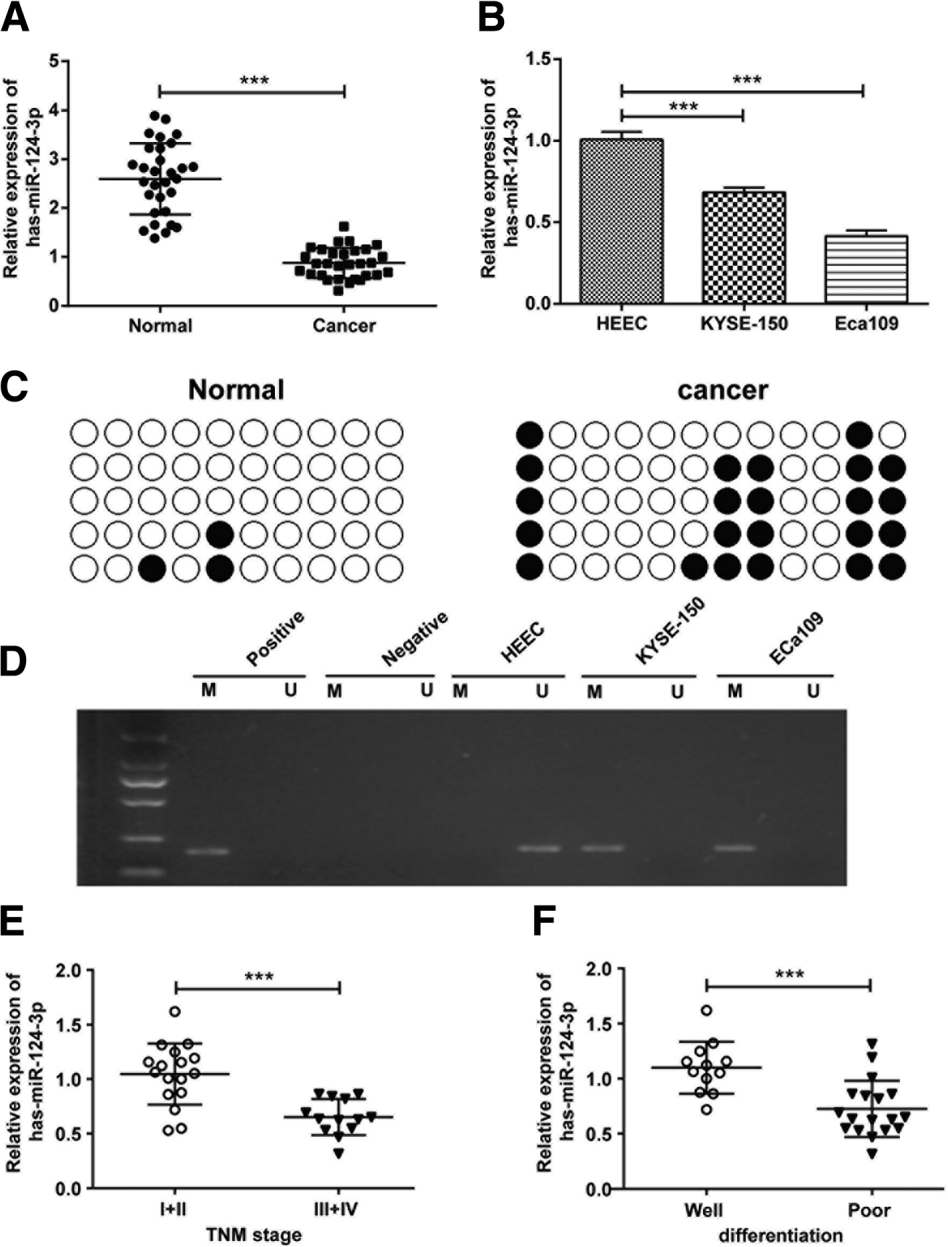
The expression and hypermethylation of hsa-miR-124-3p in ESCC tissues and cells. The mRNA expression of hsa-miR-124-3p in normal tissues and ESCC tissues (**a**) and its expression in HEEC, KYSE-150 and Eca109 cells (**b**) were determined by qRT-PCR analysis. The methylation status of hsa-miR-124-3p in normal tissues and ESCC tissues (**c**) and its status in HEEC, KYSE-150 and Eca109 cells (**d**) were determined by bisulfite Sanger sequencing and methylation specific PCR respectively. hsa-miR-124-3p expression in tumor tissues was shown in the patents with different TNM stages (**e**) and different differentiation (**f**). Experiments were performed in triplicate and each value represents mean ± SD. ^***^P < 0.001

Considering that MIR124–2 gene has been reported to be of high methylation in ESCC, we supposed that the mRNA expression of hsa-miR-124-3p, as a mature sequence, was influenced in ESCC tissues and cells. As indicated in Fig. 1a, hsa-miR-124-3p mRNA expression was significantly decreased in ESCC tissues compared with adjacent normal tissues. Meanwhile, its expression was also attenuated in ESCC cell line KYSE-150 and Eca109 cells compared with noncancerous HEEC cells (Fig. 1b). Along the lines of reduced hsa-miR-124-3p mRNA expression, we also observed that the incidence of hsa-miR-124-3p hypermethylation in ESCC tissues was significantly higher than in normal tissues (Fig. 1c). Then, as shown in Fig. 1d, hypermethylation of hsa-miR-124-3p was obvious in KYSE-150 and Eca109 cells, but not in normal HEEC cells. In addition, the clinical data indicated that low-level expression of hsa-miR-124-3p was associated with tumor node metastasis (TNM) stage III-IV and poor differentiation grade (Fig. 1e and f). However, high expression of hsa-miR-124-3p was observed in the patients with TNM stage I-II and well-differentiation grade. Therefore, there might be significant associations between hsa-miR-124-3p level and TNM stage, and differentiation grade.

### Hsa-miR-124-3p targets 3’UTR region of BCAT1 mRNA

**Fig. 2:**
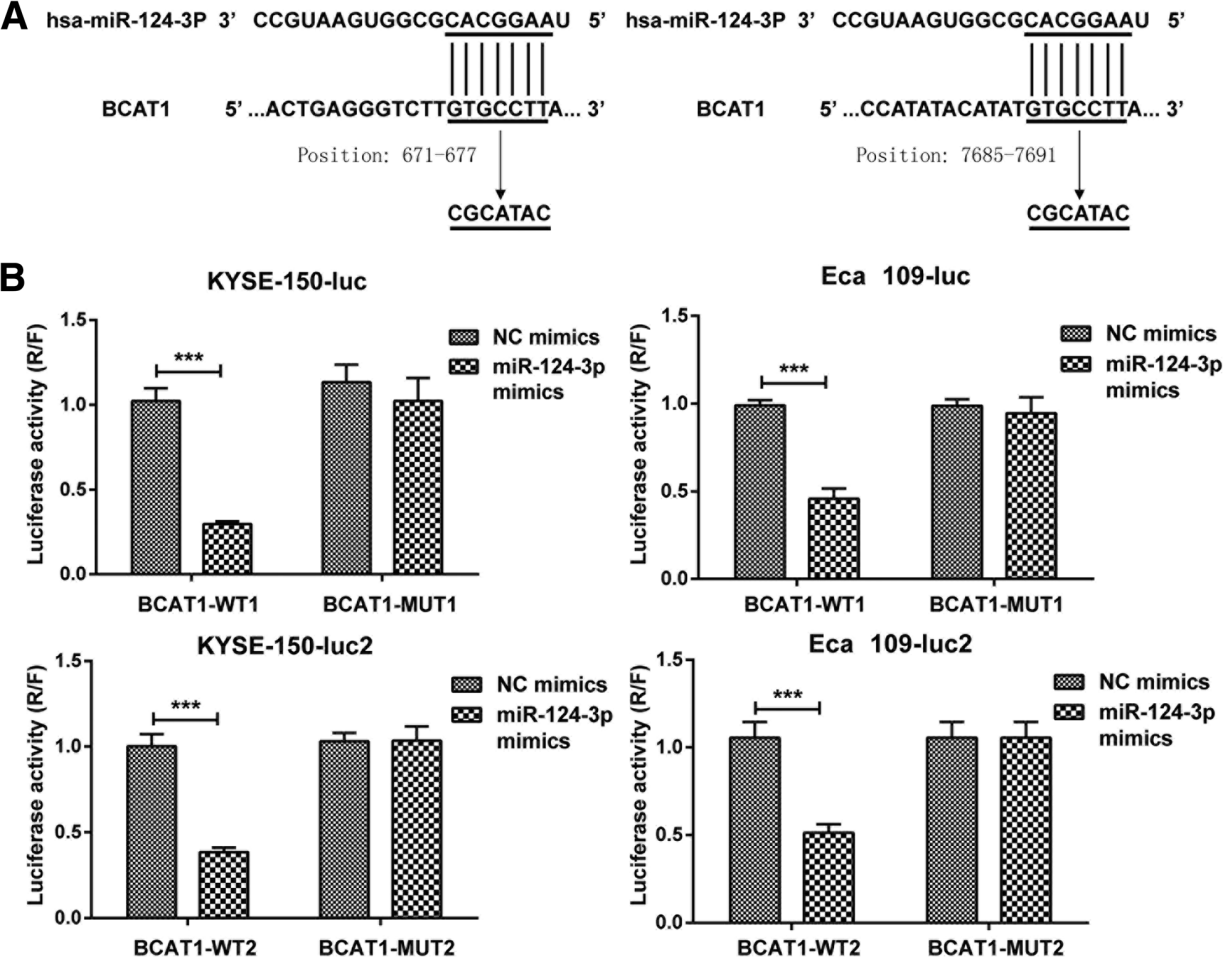
hsa-miR-124-3p targets to 3′-UTR region of BCAT1 mRNA. **a** Bioinformatic analysis shows that hsa-miR-124-3p binds to position 671–677 (WT1) and position 7685–7691 (WT2) of BCAT1 of BCAT1 3′-UTR region through base complementary pairing. MUT1 refers to the mutant sequence of WT1 and MUT2 is the mutant sequence of WT2. **b** The combination of hsa-miR-124-3p and BCAT1 was confirmed by a dual-luciferase reporter assay. KYSE-150 or Eca109 cells were co-transfected with psi-CHECK2-BCAT1–3′-UTR (WT1/2 or MUT1/2) and miR-124-3p mimics (or NC mimics). The luciferase activity was calculated as the ratio of renilla luciferase activity to firefly luciferase after 48 h. The experiments were performed in triplicate and each value represents mean ± SD. ***p < 0.001

To determine the target gene of hsa-miR-124-3p, we used bioinformatics software of Miranda to predict its downstream target gene. The data showed that 5′-UTR region of hsa-miR-124-3p could bind to 3′-UTR region of BCAT1 gene with two potential target sites of 671–677 and 7685–7691 (Fig. 2a), which has been associated with cell proliferation, migration and invasion [17, 18]. Subsequently, a dual-luciferase reporter assay was performed to confirm that BCAT1 mRNA is bona fide target of hsa-miR-124-3p. The data demonstrated that the relative luciferase activity of the BCAT1-WT1 3’UTR reporter was significantly inhibited in KYSE-150 and Eca109 cells with treatment of miR-124-3p mimics compared to the cells transfected with NC mimics. In contrast, the luciferase activity of BCAT1-MUT1 3’UTR reporter with the mutant binding site was unaffected by co-transfection of miR-124-3p mimics compared with NC mimics co-transfected. Similarly, the combination between BCAT1-WT2 3’UTR and hsa-miR-124-3p was also proved through dual-luciferase reporter assay (Fig. 2b).

### BCAT1 is upregulated in ESCC tissues and cells and shows association with TNM stage, differentiation grade and survival

**Fig. 3:**
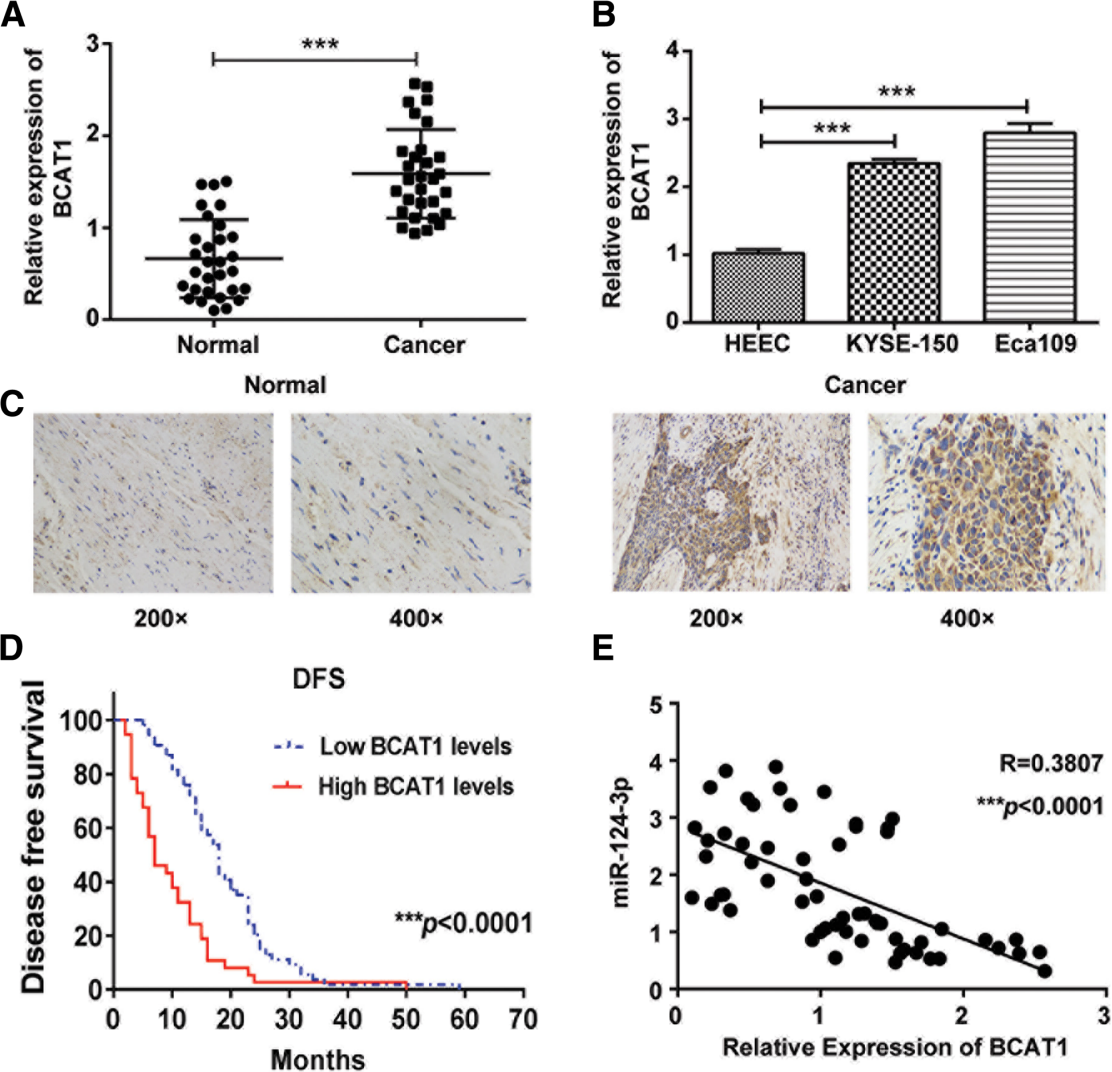
The expression of BCAT1 in ESCC tissues and cells. The mRNA expression of BCAT1 in normal tissues and ESCC tissues (**a**) and its expression in HEEC, KYSE-150 and Eca109 cells (**b**) were determined by qRT-PCR analysis. **c** The protein expression of BCAT1 in normal tissues and ESCC tissues was determined by immunohistochemistry staining analysis. **d** The Kaplan-Meier survival curve of ESCC patients with different levels of BCAT1 expression. **e** The correlation between hsa-miR-124-3p and BCAT1 gene expression in ESCC tissues was assessed by qRT-PCR (*n* = 50; *p* < 0.0001). The experiments were performed in triplicate and each value represents mean ± SD. *p < 0.05, ***p < 0.001

As BCAT1 was predicted to be the downstream target gene of hsa-miR-124-3p, we further studied the expression of BCAT1 in ESCC tissues and cells. As expected, the mRNA expression of BCAT1 was markedly increased in ESCC tissues compared with normal tissues (Fig. 3a). Meanwhile, its expression was also upregulated in KYSE-150 and Eca109 cells compared with HEEC cells (Fig. 3b). In agreement with the increased mRNA of BCAT1, BCAT1 protein expression was also significantly increased in ESCC tissues through the immunohistochemistry staining assay (Fig. 3c). Furthermore, the clinical data showed that BCAT1 protein expression level in ESCC was negatively associated with disease-free survival (Fig. 3d). In addition, the clinical data indicated that the expression of BCAT1 was significantly associated with tumor size, pathologic stage, T classification and differentiation grade of ESCC patients. However, there was no significant correlation between the expression level of BCAT1 and age, sex or N classification of ESCC patients (Table 1). Furthermore, the data demonstrated that miR-124-3p was of negative correlation with BCAT1 gene in ESCC tissues (Fig. 3e).

**Table 1:** Clinicopathologic correlation of BCAT1 expression in ESCC cancer

Factors	No. of patients (%)		
	BCAT1 low expression	BCAT1 high expression	p
Age (y)			0.9626
≥ 59	28	19	
<59	26	18	
Sex			0.2141
Male	41	32	
Female	13	5	
Tumor size (cm)			*0.0499
≥ 3	30	28	
<3	24	9	
Pathologic stage (pTNM)			*0.0113
I	10	1	
II	29	16	
III	15	20	
T stage			*0.0233
1	9	1	
2	12	6	
3	31	28	
4	2	2	
N stage			0.1423
0	37	19	
1	14	12	
2	3	6	
Differentiation grade			*0.0320
Well	9	3	
Moderate	28	14	
Poor	17	20	

### miR-124-3p mimics inhibits proliferation and migration of ESCC cells

**Fig. 4:**
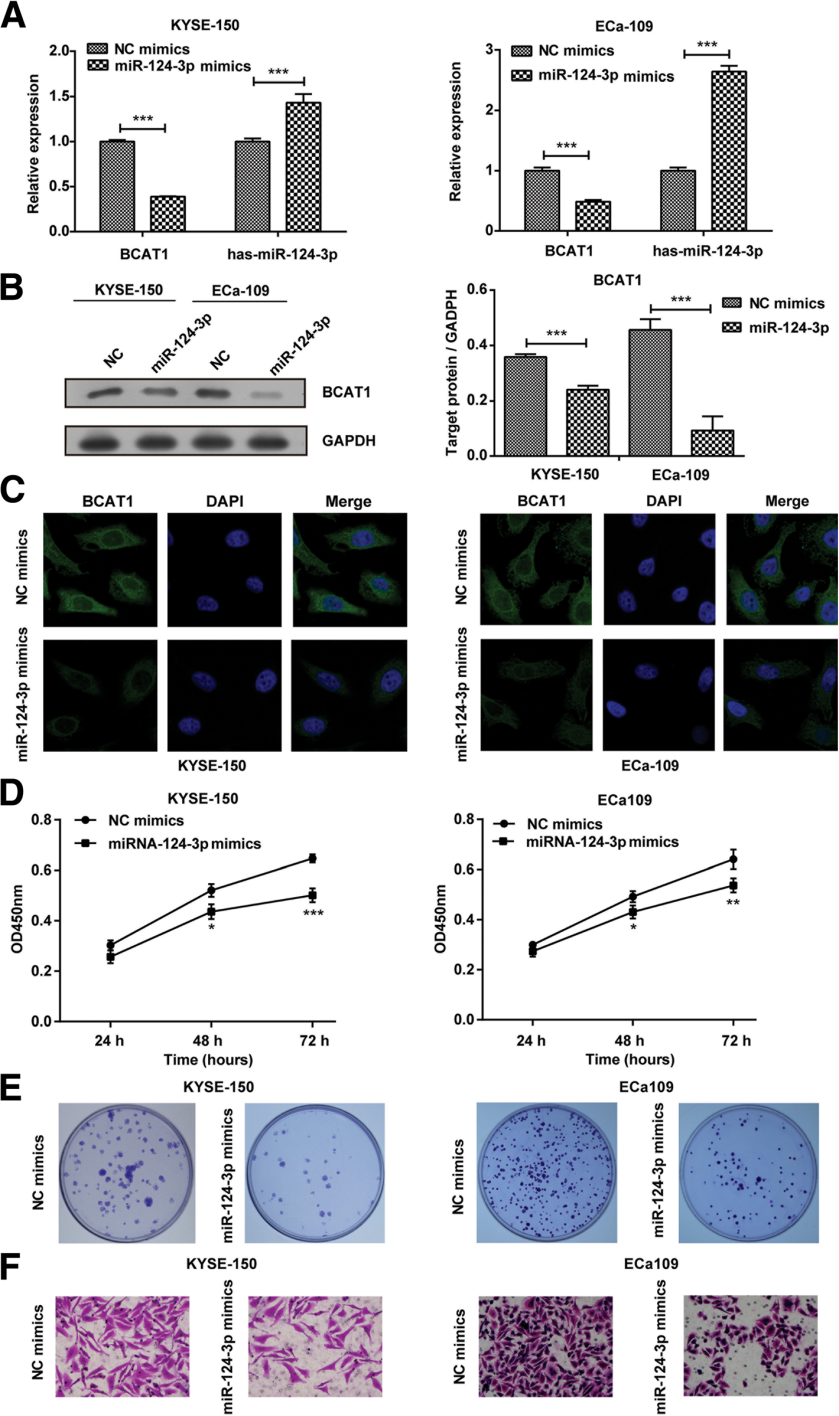
The effect of miR-124-3p mimics on cell proliferation and migration of ESCC cells. **a** The mRNA expression of BCAT1 and hsa-miR-124-3p in KYSE-150 and Eca109 cells transfected with miR-124-3p mimics or NC mimics were determined by qRT-PCR. **b-c** The protein expression of BCAT1 in KYSE-150 and Eca109 cells transfected with miR-124-3p mimics or NC mimics were detected by western blotting assay (**b**) and immunofluorescence staining assay (**c**). The target protein expression relative to GAPDH expression was displayed on the right of Fig. 4b. **d-e** The effect of miR-124-3p mimics on KYSE-150 and Eca109 cells proliferation was detected by CCK8 assay (**d**) and colony formation assay (**e**). **f** The effect of miR-124-3p mimics on KYSE-150 and Eca109 cells migration was detected by transwell assay. The experiments were performed in triplicate and each value represents mean ± SD. ^*^P < 0.05, ^**^P < 0.01, ^***^P < 0.001

To clarify miR-124-3p/BCAT1-mediated protection in ESCC, we first treated KYSE-150 or Eca109 cells with miR-124-3p mimics and NC mimics. As indicated in Fig. 4a, miR-124-3p mimics induced the mRNA expression of hsa-miR-124-3p and attenuated the mRNA expression of BCAT1 in KYSE-150 and Eca109 cells compared with NC mimics. At the same time, miR-124-3p mimics also inhibited the protein expression of BCAT1 in KYSE-150 and Eca109 cells through western blotting analysis (Fig. 4b), which is in line with the result of immunofluorescence staining analysis (Fig. 4c). Next, we detected the effect of miR-124-3p mimics on proliferation and migration of ESCC cells. KYSE-150 and Eca109 cells treated with miR-124-3p mimics showed decreased cell viability compared to cells with NC mimics after 48 h and 72 h (Fig. 4d). Meanwhile, the treatment of miR-124-3p mimics resulted in decreased colony formation in KYSE-150 and Eca109 cells (Fig. 4e). In addition, the cell migration of two ESCC cells indicated by transwell assay were significantly inhibited by miR-124-3p mimics transfection compared with NC mimics transfection (Fig. 4f). These results indicated that increased hsa-miR-124-3p might critically contribute to the inhibition of cell proliferation and migration in ESCC, which might be associated with the decreased expression of BCAT1.

### Inhibition of proliferation and migration induced by hsa-miR-124-3p was associated with the decreased BCAT1 expression

**Fig. 5:**
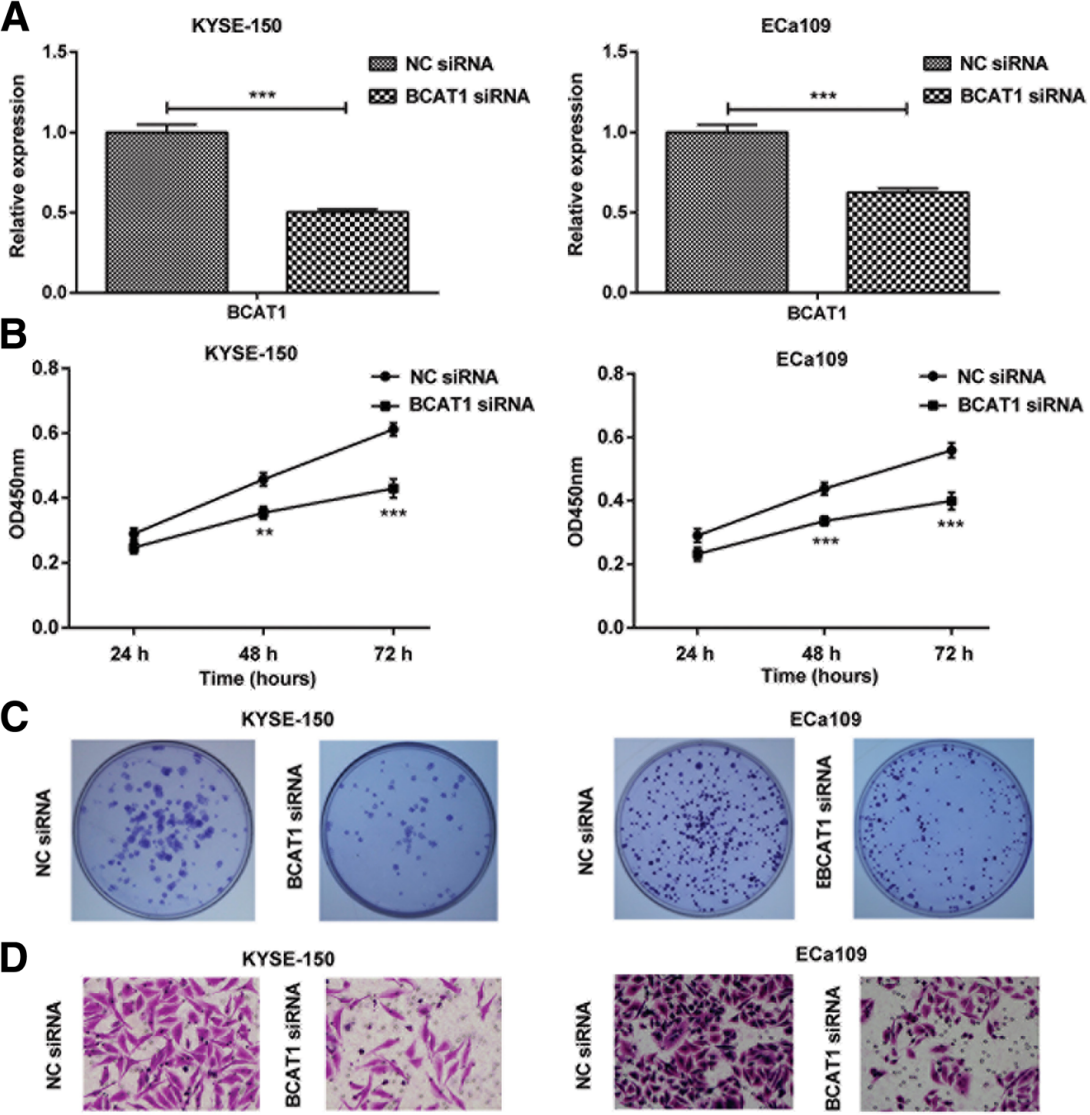
The effect of BCAT1 siRNA on proliferation and migration of ESCC cells. **a** The mRNA expression of BCAT1 in KYSE-150 and Eca109 cells transfected with BCAT1 siRNA or NC siRNA were determined by qRT-PCR. **b-c** The effect of BCAT1 siRNA on KYSE-150 and Eca109 cells proliferation was detected by CCK8 assay (**b**) and colony formation assay (**c**). **d** The effect of BCAT1 siRNA on KYSE-150 and Eca109 cells migration was detected by transwell assay. The experiments were performed in triplicate and each value represents mean ± SD. ^*^P < 0.05, ^**^P < 0.01, ^***^P < 0.001

To determine whether hsa-miR-124-3p inhibition of cell proliferation and migration was associated with BCAT1 expression, we firstly explore the effect of BCAT1 on the proliferation and migration of ESCC cells. KYSE-150 and Eca109 cells were first transfected with BCAT1siRNA and NC siRNA. The results showed that compared to NC siRNA, BCAT1 siRNA treatment inhibited mRNA expression of BCAT1 in two ESCC cells (Fig. 5a). Similar to the effect of miR-124-3p mimics transfection, knockdown of BCAT1 significantly inhibited cell viability (Fig. 5b), colony formation (Fig. 5c) and cell migration (Fig. 5d) in KYSE-150 and Eca109 cells. The result implies that BCAT1 is positively associated with proliferation and migration of ESCC cells.

**Fig. 6:**
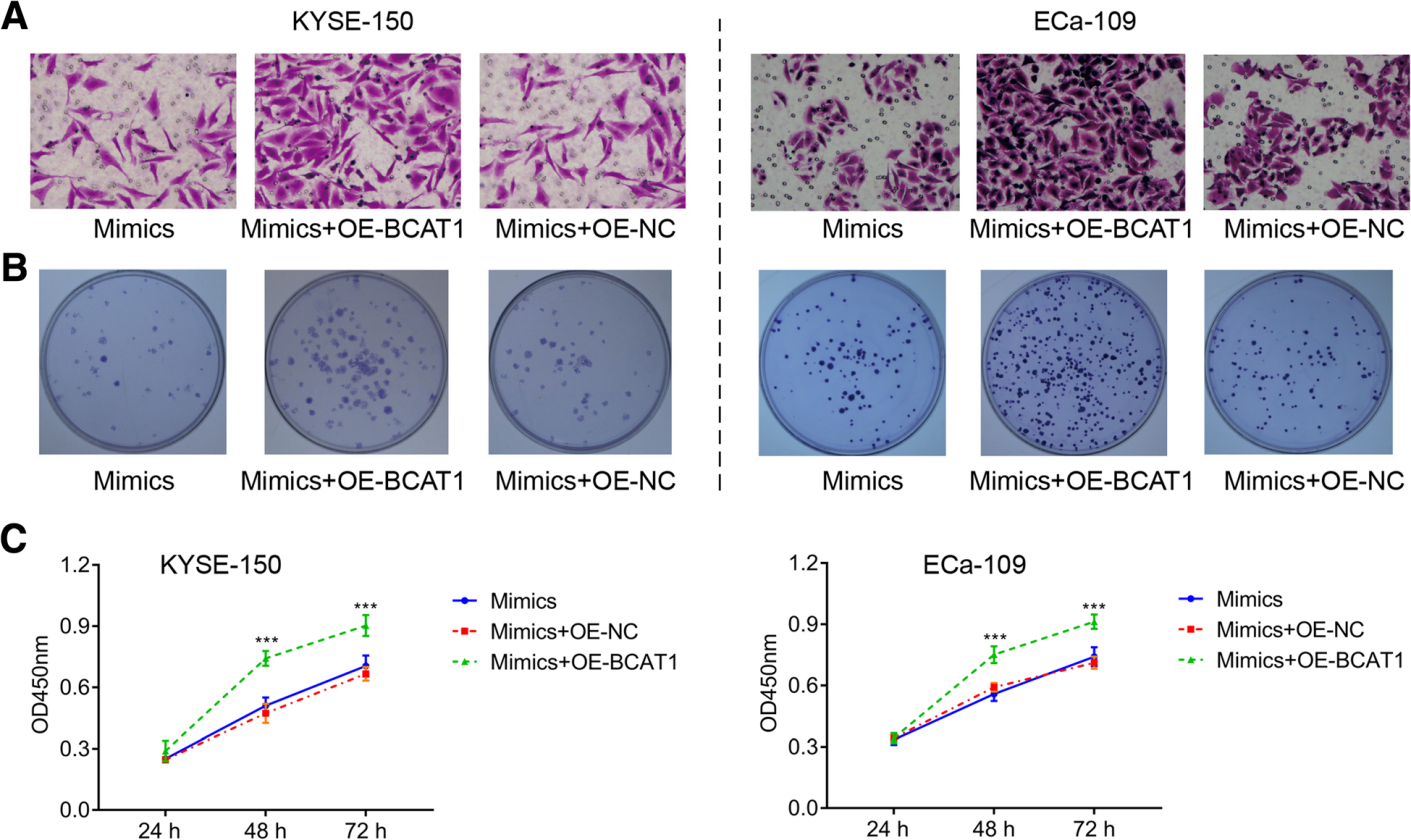
The effect of BCAT1 overexpression on proliferation and migration of ESCC cells treated with hsa-miR-124-3p mimics. KYSE-150 or Eca109 cells were transfected with OE-NC, OE-BACT1 or solvent control in the presence of hsa-miR-124-3p mimics. **a** The migration of KYSE-150 and Eca109 cells was detected by transwell assay. **b**-**c** The proliferation of KYSE-150 and Eca109 cells was detected by colony formation (**b**) assay and CCK8 assay (**c**). The experiments were performed in triplicate and each value represents mean ± SD. ***P < 0.001

Next, to confirm that hsa-miR-124-3p inhibition of cell proliferation and migration depends on BCAT1 expression. KYSE-150 or Eca109 cells were transfected with OE-NC, OE-BACT1 or solvent control in the presence of hsa-miR-124-3p mimics. The results showed that BACT1 overexpression (OE-BACT1) enhanced migration (Fig. 6a) and proliferation (Fig. 6b-c), compared to the group OE-NC and solvent control. These finding suggests that hsa-miR-124-3p-medaited inhibition of proliferation and migration depends on the expression of BCAT1.

### DNMT1 regulates hsa-miR-124-3p / BCAT1 in ESCC cells

**Fig. 7:**
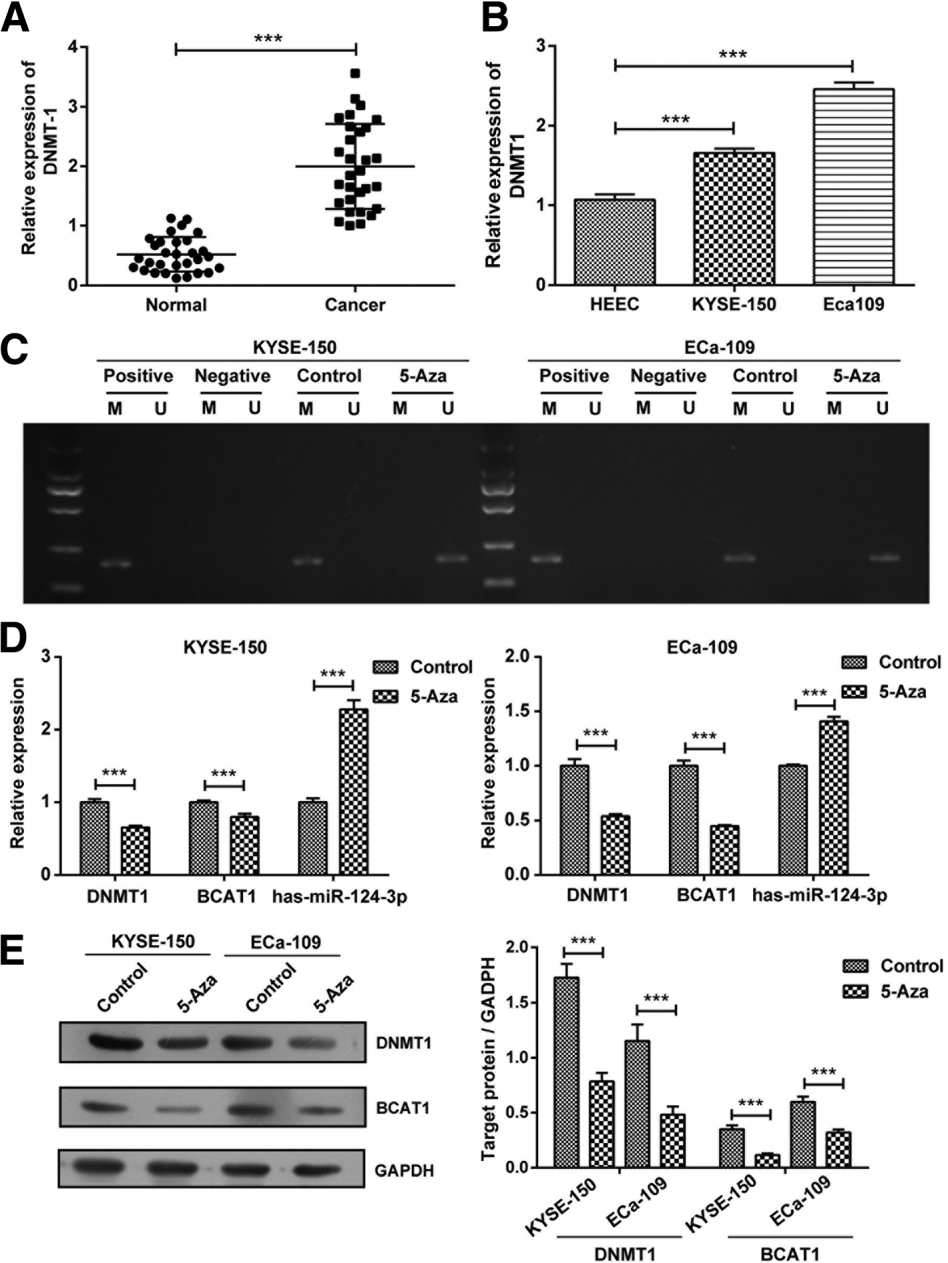
The regulatory role of DNMT1 inhibitor on expression of hsa-miR-124-3p and BCAT1 in ESCC cells. **a**-**b** The mRNA expression of DNMT1 in normal tissues and ESCC tissues (**a**) and its expression in HEEC, KYSE-150 and Eca109 cells (**b**) were determined by qRT-PCR analysis. **c** The methylation status of hsa-miR-124-3p in KYSE-150 and Eca109 cells treated with DNMT1 inhibitor 5 μM 5-Azacitidine (5-Aza) or its control (PBS), was detected by methylation specific PCR. **d** The mRNA expression of DNMT1, hsa-miR-124-3p and BCAT1 in KYSE-150 and Eca109 cells treated with 5-Aza or PBS were determined by qRT-PCR analysis. **e** The protein expression of DNMT1 and BCAT1 were detected by western blotting assay. The target protein expression relative to GAPDH expression was displayed on the right. The experiments were performed in triplicate and each value represents mean ± SD. ^***^P < 0.001

As DNMT1 plays an important role in mediating the methylation of microRNAs in cancer cells [21, 22], we investigated whether it was involved in the regulation of hsa-miR-124-3p / BCAT1 in ESCC. Firstly, we found that DNMT1 mRNA expression was markedly increased in ESCC tissues (Fig. 7a) as well as two ESCC cell lines (KYSE-150 and Eca109 cells) (Fig. 7b). Next, as expected, the hsa-miR-124-3p was demethylated after treatment with 5 μM 5-Azacitidine (5-Aza), the inhibitor of DNMT1, in KYSE-150 and Eca109 cells, which means DNMT1 contributes to the methylation of hsa-miR-124-3p (Fig. 7c). Furthermore, 5-Aza treatment significantly promoted the mRNA expression of hsa-miR-124-3p and attenuated the mRNA expression of BCAT1 in KYSE-150 and Eca109 cells (Fig. 7d). Meanwhile, it attenuated the protein expression of BCAT1 in these two ESCC cells as well (Fig. 7e).

**Fig. 8:**
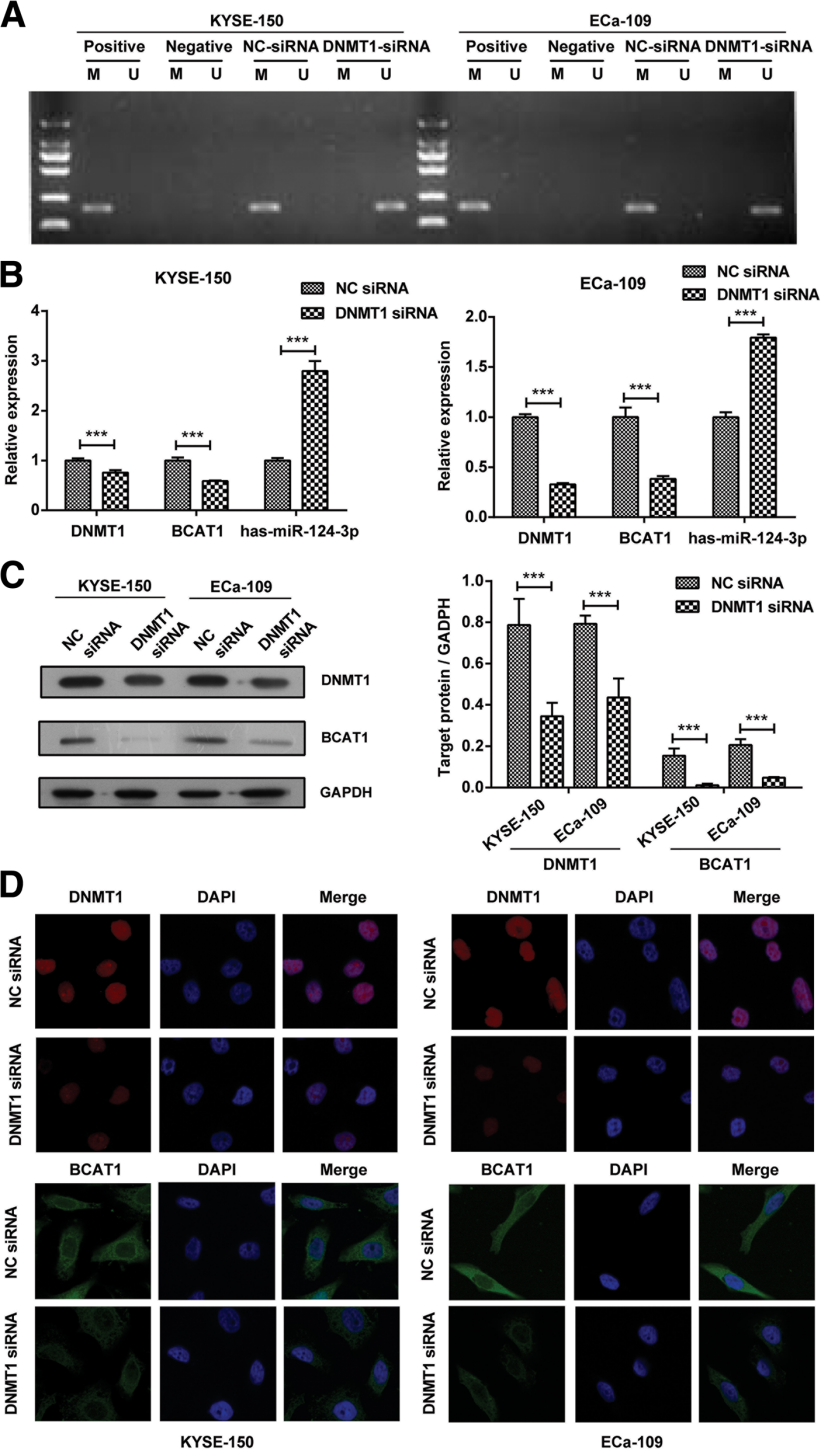
The regulatory role of DNMT1 siRNA on hsa-miR-124-3p / BCAT1 in ESCC cells. **a** The methylation status of hsa-miR-124-3p in KYSE-150 and Eca109 cells transfected with DNMT1 siRNA or NC siRNA was detected by methylation specific PCR. **b** The mRNA expression of DNMT1, hsa-miR-124-3p and BCAT1 in KYSE-150 and Eca109 cells transfected with DNMT1 siRNA or NC siRNA were determined by qRT-PCR analysis. **c**-**d** The protein expression of DNMT1 and BCAT1 were detected by western blotting assay (**c**) and immunofluorescence staining assay (**d**) respectively. The target protein expression relative to GAPDH expression was displayed on the right of Fig. 8c. The experiments were performed in triplicate and each value represents mean ± SD. ^***^P < 0.001

In order to further confirm the regulatory role of DNMT1 to hsa-miR-124-3p / BCAT1 in ESCC cells, we treated KYSE-150 and Eca109 cells with DNMT1 siRNA. The data revealed that DNMT1 siRNA induced the demethylation of hsa-miR-124-3p and further promoted its mRNA expression in KYSE-150 and Eca109 cells (Fig. 8a and b). Accordingly, we found that the mRNA and protein expression of BCAT1 were both inhibited after treatment with DNMT1 siRNA compared with NC siRNA treatment (Fig. 8b-d). Collectively, these findings indicate that DNMT1 is essential for the methylation of hsa-miR-124-3p and subsequently induce the expression of BCAT1, which involves in the cell proliferation and migration of ESCC cells.

## Discussion

MiR-124 has been shown to function as a tumor suppressor in esophageal cancer [12]. However, it is not clear concerning the mechanism by which miR-124 expression was downregulated in esophageal cancer and its subtype ESCC. In addition, there is still lack of direct experiment evidence suggesting that miR-124 as a tumor suppressor regulates the initiation and development of ESCC. We herein showed that significantly increased expression of hsa-miR-124-3p was observed in the paracancerous normal tissues of ESCC and the normal cell line HEEC compared to ESCC tissues and two ESCC cell lines. In addition, over-expression of hsa-miR-124-3p is highly negatively correlated with proliferation and migration in several ESCC cells in the present study. These findings imply that decreased expression of hsa-miR-124-3p might be highly correlated with initiation and progression of ESCC.

The first crucial insight gleaned from our present study is that although miR-124 has been reported to target several associated-cancer genes including RAC1, ROCK1 [23], STAT3, the androgen receptor [24], PIK3CA [25], EZH2 [26] and CBL [27], we showed a new target BCAT1 gene of hsa-miR-124-3p through bioinformatics prediction and dual-Luciferase reporter assay. The BCAT1 gene, known previously as ECA39, is an enzyme transforming α-amino groups of branched chain amino acids (BCAAs) to a-ketoglutarates (a-KG) [15, 16], yielding glutamate frequently required for the growth of malignant cells [28]. BCAT1 has been shown overexpression in some human malignancies, which is highly correlated with initiation and progression of malignant tumor [17, 18, 29]. However, to the best of our knowledge, there is no report showing association of BCAT1 overexpression with ESCC. We thus speculated that growth and migration inhibition in ESCC cells mediated by hsa-miR-124-3p was associated with downregulation of BCAT1 expression. Consistent with this, our results indicated that BCAT1 knockdown significantly inhibited cell proliferation and migration of ESCC cell lines KYSE-150 and Eca109.

Regulation of BCAT1 expression in different tumors involves in different mechanisms. The aberrant DNA methylation in *BCAT1* promotor region was associated with in colorectal cancer, ovarian cancer and gliomas [17, 30]. These findings suggest that epigenetic mechanisms could account for altered BCAT1 expression in different cancer types, including EOC. In the present study, however, we showed that BCAT1 expression was directly regulated by hsa-miR-124-3p since hsa-miR-124-3p bound to 3′-UTR region of BCAT1 gene, degrading BCAT1 mRNA. In fact, we also observed downregulation of BCAT1 expression in KYSE-150 and Eca109 cells treated with hsa-miR-124-3p mimics. It should be also noted that BCAT1 expression might be also regulated by DNA methylation in BCAT1 promotor region in KYSE-150 and Eca109 cells although we did not provide the direct evidence. This is because expression of DNMT1, an enzyme that catalyzes the transfer of methyl groups to specific CpG structures in DNA, significantly increased in ESCC tissues and two ESCC cell lines.

Some miRNAs containing CpG islands are susceptible to methylation-associated silencing [31–33]. Methylation-associated silencing of tumor-suppressive miRNAs might play a crucial role in the tumorigenesis through activating oncogenic pathways. MiR-124, as a typical tumor-suppressive miRNA, has also been found epigenetically silenced in cholangiocarcinoma, cervical cancer and pancreatic cancer [34–36]. Similarly, a recent study showed that miR-124 gene were highly methylated in LNM-positive ESCC [10]. Those findings are in agreement with our results showing hypermethylation miR-124 gene in ESCC tissues and two ESCC cell lines. However, in the previous study it is unclear that hypermethylation in miR-124 gene was linked to overexpression of DNMT in ESCC. In the present study, we provide the direct evidence suggesting that hypermethylation in miR-124 gene was strongly mediated by DNMT1 through DNMT1 knockdown and 5-AZ treatment. This finding is consistent with the previous study showing DNMT1-mediated downregulation of miR-124 expression in the intrahepatic cholangiocarcinoma [34]. Furthermore, our results demonstrated that DNMT1 knockdown or 5-AZ treatment significantly inhibited cell proliferation and migration of ESCC cell lines KYSE-150 and Eca109 through increasing hsa-miR-124-3p expression and inhibiting downstream BCAT1 expression.

## Conclusions

In summary, our present work indicates that low hsa-miR-124-3p levels mediated by DNMT1 promote ESCC cell proliferation and invasion by targeting BCAT1, suggesting that DNMT1/miR-124/ BCAT1 axis plays an important role in regulating development and progression of ESCC. These findings suggest that inhibitors against the activity of DNMT1 and/or BCAT1 might be a novel targeted therapeutic choice against ESCC.

## Data Availability

The data used and analyzed during this study are available from the corresponding author on request.

## Abbreviations

5′-Aza: 5′-Azacitidine
BCAAs: Branched chain amino acids
BCAT1: Branched chain amino acid transaminase 1
CCK-8: Cell Counting Kit-8
DAPI: 4,6-diamino-2-phenyl indole
DNMTs: DNA methyltransferases
ESCC: Esophageal squamous cell carcinoma
FITC: Fluoresceine isothiocyanate
GAPDH: Glyceraldehyde-3-phosphate dehydrogenase
HOXB2: Homeobox protein Hox-B2
MiRNAs: microRNAs
MSP: Methylation Specific PCR
NC: Negative control
NEFL: Neurofilament light polypeptide
OBSL1: Obscurin-like 1
SLC15A3: Solute carrier family 15 member 3
TNM: Tumor Lymph Node Metastasis
UTR: Untranslated Region
